# Recognizing Emotions through Facial Expressions: A Largescale Experimental Study

**DOI:** 10.3390/ijerph17207420

**Published:** 2020-10-12

**Authors:** Artemisa R. Dores, Fernando Barbosa, Cristina Queirós, Irene P. Carvalho, Mark D. Griffiths

**Affiliations:** 1 Center for Rehabilitation Research, School of Health, Polytechnic of Porto, 4200-072 Porto, Portugal; 2 Laboratory of Neuropsychophysiology, Faculty of Psychology and Education Sciences, University of Porto, 4200-135 Porto, Portugal; fbarbosa@fpce.up.pt; 3 Faculty of Psychology and Education Sciences, University of Porto, 4200-135 Porto, Portugal; cqueiros@fpce.up.pt; 4 Clinical Neurosciences and Mental Health, School of Medicine, University of Porto, 4200-319 Porto, Portugal; irenec@med.up.pt; 5 International Gaming Research Unit, Psychology Department, Nottingham Trent University, Nottingham NG1 4FQ, UK; mark.griffiths@ntu.ac.uk

**Keywords:** emotions, emotion recognition, facial expressions, RaFD, gender differences

## Abstract

Experimental research examining emotional processes is typically based on the observation of images with affective content, including facial expressions. Future studies will benefit from databases with emotion-inducing stimuli in which characteristics of the stimuli potentially influencing results can be controlled. This study presents Portuguese normative data for the identification of seven facial expressions of emotions (plus a neutral face), on the Radboud Faces Database (RaFD). The effect of participants’ gender and models’ sex on emotion recognition was also examined. Participants (*N* = 1249) were exposed to 312 pictures of white adults displaying emotional and neutral faces with a frontal gaze. Recognition agreement between the displayed and participants’ chosen expressions ranged from 69% (for anger) to 97% (for happiness). Recognition levels were significantly higher among women than among men only for anger and contempt. The emotion recognition was higher either in female models or in male models depending on the emotion. Overall, the results show high recognition levels of the facial expressions presented, indicating that the RaFD provides adequate stimuli for studies examining the recognition of facial expressions of emotion among college students. Participants’ gender had a limited influence on emotion recognition, but the sex of the model requires additional consideration.

## 1. Introduction

The conventional method for studying emotional perception and recognition typically consists of presenting facial expressions of emotions in laboratory experiments. However, the parameters of the available stimuli do not always correspond to the objectives of the studies [1]. For example, the low control that researchers have over emotional stimuli can cause methodological artifacts that affect emotion recognition [2,3,4,5,6]. The use of databases containing faces as emotional stimuli and enabling researchers to monitor technical features such as the background image, and models’ features such as facial expression or sex, constitutes a positive contribution to the field. This material can also have practical applications in other domains. For example, difficulty recognizing emotions has been associated with various psychological disorders, such as depression [7,8,9], attention deficit hyperactivity disorder [10,11,12,13,14], bipolar personality disorder [15], eating disorders [16], Williams syndrome [17], schizophrenia [18], and autism spectrum disorders [19], amongst others. Again, the aforementioned applications require the development of sets of standardized stimuli and their validation, including normative data for different countries and cultures. Basic emotions theory has limitations, and evidence shows that the facial expressions of emotion are not as universal as initially thought. Even “basic” emotions may lack a uniform affective meaning across and within societies, with its recognition being influenced by cultural factors. Recent studies of emotion perception conducted in small-scale societies show evidence of human diversity [20], which has received support from the behavioral, cognitive, and biological sciences [21,22,23,24]. 

Although this recent research needs to be taken into consideration, facial expressions are, according to Ekman, both universal (at least some part) and culture specific. This perspective informs the current study, justifying the need for research that examines how the Radboud Faces Database (RaFD) behaves in different countries [25]. 

Different databases of faces with varying emotional expressions are currently available, such as the Japanese and Caucasian Facial Expressions of Emotion/Japanese and Caucasian Neutral Faces Collection (JACFEE /JACNeuF) [26], the Montreal Set of Facial Displays of Emotion (MSFDE) [27], the Amsterdam Dynamic Facial Expression Set (ADFES) [28], the Facial Expression Subset [29], the NimStim Face Stimulus Set (NimStim) [30], the Karolinska Directed Emotional Faces (KDEF) [31], and the Radboud Faces Database (RaFD) [32] (for a review see [33]). The RaFD is one of the most recently established databases, and its quality has justified further studies concerning its use. The RaFD is a free database for non-commercial scientific research that comprises 49 white male, female, and child models, and 18 Moroccan male models. Each model was trained to present seven emotional expressions (anger, sadness, fear, disgust, surprise, happiness, and contempt) and a neutral expression. This training was based on the Facial Action Coding System (FACS) [34]. The photographs of the models were taken from five simultaneous camera angles, and all the emotional expressions have three gaze directions (direct, shift to the left, and shift to the right) in a total of 120 pictures per model [32] (for more information, see www.rafd.nl). 

Due to its characteristics, the RaFD is a resource with research potential in areas where the use of emotional facial expressions is important, such as facial recognition and social interaction. This database also provides a large number of models, helping to prevent habituation effects [35]. It also allows for the control of features such as gaze direction and camera angle. Technical characteristics such as focal distance, clothing, lighting conditions, and background image are also controlled across photographs. 

The role of static versus more ecological stimuli has been presented in the literature [36]. The fact that models have different ages, sexes, and ethnicities, and the fact that the pictures are in color increase the database’s ecological validity with respect to these aforementioned variables. Another advantage of the RaFD is that it provides a neutral expression in addition to the seven primary emotions. This is useful, for example, in functional neuroimaging studies in which a baseline is necessary for comparison purposes [37,38]. Additionally, for each depicted expression, data are provided concerning its intensity, clarity, or genuineness, besides the overall valence. The fact that the RaFD provides models of both sexes allows comparative research investigating emotion recognition among male and female models. Interestingly, a study by Langner et al. (2010) reported that the facial expressions of female models were better recognized than those of male models [32]. Data on the validation of photographs taken by the original authors from a camera angle of 90° reinforce the adequacy of the RaFD. These data show a high level of agreement of the responses with the emotions being displayed (min agreement rate = 53%, max = 98%). In general, mean percentage agreement between presented and recognized emotions was 82% (*Mdn* = 88%, *SD* = 19%) [32]. Similar to other databases, it is possible to observe a different emotion recognition pattern between positive and negative emotions in this database, with the former being better recognized than the latter. Recognition mean scores ranged from 98% (for happiness) to 58% (for contempt). However, it should be taken into consideration that happiness is the only positive emotion available (other positive emotions such as pride, amusement, compassion, and love are not part of the database), while there are emotions of negative valence that are similar to contempt (e.g., anger), probably affecting its recognition. 

The distinction between the recognition of positive and negative emotions has been explained in the literature (amongst other possible reasons) through the interactions between cognition and emotion. For example, research has shown that processing positive emotions (e.g., happiness) requires less attentional resources than processing negative emotions (e.g., anger) [39,40,41,42,43,44,45]. Older adults also have shown slower responses to negative faces than to neutral faces, which has been viewed as indicative of their avoidance of negative information [46].

Taking advantage of its characteristics, various studies on emotion recognition have used the RaFD for specific purposes [47,48,49,50,51]. Recently there have been increased efforts to validate this database with different populations, such as Indian participants [52] and children [53], both with good results. In the first case, despite the significant cross-cultural differences in the classification of emotions that should be taken in account, the authors found high recognition agreement rates [52], which were similar to the ones reported in the original study by Langner et al. (2010) [32]. In the second case, children’s emotion recognition pattern was identical to the adults’ pattern, although children were less able to distinguish between similar emotions [54]. In another study, Dawel et al. (2017) pointed out that an advantage of the RaFD database was the availability of genuineness ratings [55]. However, using the new method for rating perceived genuineness (i.e., using a neutral-midpoint scale) led to some of the RaFD expressions being perceived as fake [55]. 

The availability of databases with different emotional stimuli, such as the RaFD, is also important to study sex differences in the recognition of emotions. The literature suggests that sex affects recognition of facial expressions of emotion. For example, a meta-analysis showed “a small overall advantage in favor of female participants on emotion recognition tasks (*d* = 0.19). However, the magnitude of the difference between women and men was moderated by several factors, namely specific emotions, emotion valence (negative, positive), sex of the actor, sensory modality (visual, audio, audio-visual) and participants’ age” [56]. A sex effect was also found in a very recent study with older adults, in which female participants recognized emotions better than their male counterparts [53].

Among other factors, attention and related brain networks appear to play an important role the comprehension of sex differences in emotion recognition. For example, Gupta (2012) advocated the role of attention and evaluation in distinct neural systems for men and women during emotional processing [57]. It has been suggested that female participants show remarkable attention and evaluative bias even for the processing of moderately negative stimuli, whereas men do not show such bias [58,59,60]. Future work on the neural processing of emotional information, might benefit from the validation of proper databases.

Although research shows the adequacy of RaFD’s facial expressions for emotion recognition tasks, its use in different cultures and countries, either for research or other purposes (e.g., clinical), demands the development of specific normative data. The present study contributes to this endeavor, by testing the RaFD on a largescale Portuguese sample, using the seven emotions plus the neutral one available in this database, and evaluating sex differences in emotion recognition. More specifically, the aims of the study were twofold: (i) to present Portuguese normative data regarding the identification of seven facial expressions of emotion (anger, sadness, fear, disgust, surprise, happiness, contempt) and a neutral expression from the RaFD, and (ii) to study the influence of the sex of the model and the sex of the participant in the performance of this task. 

## 2. Materials and Methods 

### 2.1. Participants

The present study initially comprised 1249 college students recruited from health science courses, who participated in a recognition task of facial expressions of emotion. Their mean age was 20.2 years (SD = 3.5). Most participants were women (84.4% of the sample), with a mean age of 20.1 years (SD = 3.3), and 192 were men (15.4%), with a mean age of 20.6 years (SD = 4.6). This corresponds to the typical sex distribution in health sciences courses in Portuguese universities. Three participants did not report their gender and were excluded from the analyses on the effects of participants’ gender and models’ sex on facial emotion recognition. These analyses included the remaining 1246 participants. We use the term ‘sex’ to refer to the model’s actual physical features, but the term ‘gender’ is employed when referring to the sample participants to reflect the fact that they chose their own identified category [61].

The students were from various Portuguese university programs (i.e., Psychology and Health Sciences, including Neurophysiology, Cardiopneumology, Anatomical Pathology, Environmental Health, Speech Therapy, Audiology, Occupational Therapy, and Physiotherapy). Most were in the first year of their program (593; 47.5%), 327 were in the second year (26.2%), 245 in the third year (19.6%), and 70 in the fourth year (5.6%). Fourteen participants (1.1%) did not report their academic year of study. Students were invited to participate after receiving information about the study’s goals and provided written informed consent. Participation in the study was voluntary, and data anonymity and confidentiality were assured. The study was approved by the research team’s university research ethics committee (ethical code 2811-2014) and complied with the Declaration of Helsinki.

### 2.2. Materials 

An RaFD subset with all 312 frontal-gaze adult white faces photographed with a 90°-angle camera from the RaFD’s emotional expressions [32] was presented to the students. The subset comprised 39 models displaying the seven primary emotions (anger, sadness, fear, disgust, surprise, happiness, and contempt) plus a neutral expression (i.e., 39 × 8). The models wore black t-shirts. 

### 2.3. Procedure

To minimize fatigue effects, the 312 stimuli were distributed into four blocks of 78 pictures each, containing the same instances of facial expressions (the seven emotions and the neutral face), and randomly organized within each block. A group of 307 students viewed Block I, 316 different students viewed Block II, another 324 students viewed Block III, and the remaining 299 students viewed Block IV. Participants were randomly assigned to one of the blocks and instructed to identify the emotion in each facial expression, marking their choice on the answer sheets provided. Before beginning the task, participants had the opportunity to practice with six photographs to familiarize themselves with the material and the task.

The stimuli were administered to groups of eight to 50 participants at a time, with Microsoft^®^ Office PowerPoint (Microsoft^®^, Washington, DC, United States). Within each group, pictures were presented one at a time at 10-s intervals, followed by an inter-stimulus interval of one second (black slide), during which participants identified the emotion they thought was displayed on the answer sheet. The participants can respond whenever they want during this period. According to the literature, studies can use different strategies to validate databases of faces. For example, participants might be asked to name each emotion stimulus without any reference provided concerning the emotions to be recognized (free-choice task). In some cases, participants can be highly trained in facial action units to describe the expressions presented [62]. Alternatively, participants can be asked to use a forced-choice method, which includes the presentation of response categories [63,64] and subsequent verification of the agreement between presented and recognized emotional expressions. In the present study, the latter strategy was used. This enabled the research team to take advantage of standardized techniques of data analysis and to reduce response biases. More specifically, one of its advantages was to decrease (or even eliminate) the problem of missing data, because, for each expression, participants are asked to choose among the options provided. It also generates lower variability in responses, which become more easily analyzable, codified, and computerized [65].

### 2.4. Data Analyses

Comparison between emotion recognition (mean agreement rates) in the present study and in the Langner et al.’s (2010) [32] were examined using t-test. Mean differences between emotions were examined using analysis of variance (ANOVA), considering participants’ gender and models’ sex. More specifically, a mixed repeated measures ANOVA was performed on the agreement rates (mean percentage of responses in agreement with the emotion being displayed), with gender of the participant (female, male) as the between-participants factor, and sex of the model (female, male) and emotional category (anger, sadness, fear, disgust, surprise, happiness, contempt, and a neutral expression) as within-participants factors. Possible interaction effects were also analyzed (participants’ gender × emotional category; models’ sex × motional category; participants’ gender × models’ sex). Greenhouse–Geisser corrections were applied whenever sphericity assumption was not confirmed. Post-hoc tests (Unequal N HSD and Tukey) corrected for multiple comparisons were conducted on the results that were statistically significant. Statistical analyses were performed using Statistica 12.0 (2013, StatSoft, Tulsa, OK, USA), with a significance threshold level set α = 0.05.

## 3. Results

### 3.1. Main Effects on Emotion Recognition

The overall mean agreement rate between the displayed expressions and participants’ chosen expressions was 82.0% (*SD* = 10.21%). 

Table 1 shows the comparison between the perceived emotion and the depicted emotion to these and the other emotions. The unbiased “hit-rates” per expression were: 68.4% to neutral; 55.9% to anger; 61.9% to sadness; 65.7% to fear; 68.9% to disgust; 75.0% to surprise; 93.2% to happiness, and 58.1% to contempt. The level of recognition of the eight facial expressions varied according to the emotion being expressed. Anger was the least frequently recognized expression (mean agreement rate = 67.7%, *SD* = 25.3), whereas happiness was the most frequently recognized expression (mean agreement rate = 96.8%, *SD* = 1.6) (Table 1). The mixed repeated measures ANOVA showed a main effect for emotional category, *F*(7, 8708) = 324.94, *MSE* = 0.049, *p* < 0.001, η^2^_p_ = 0.021. 

Comparing the results obtained with those of Langner et al. (2010), the confusion matrix is similar in both studies, with few exceptions (i.e., in general, the same emotions were confused in both studies). The results of the statistical comparison between the recognition of each emotion in this study and in the study by Langner et al. (2010) are presented in Table 2 [32]. 

**Figure 1:**
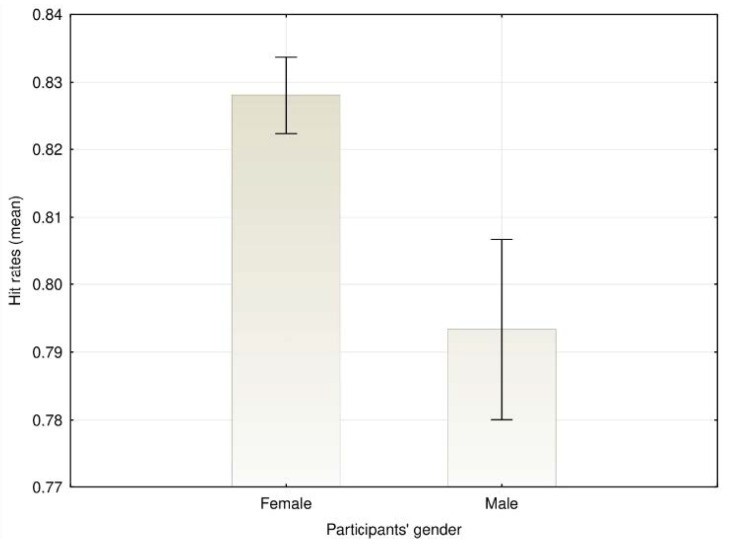
Mean agreement rates for the emotion recognition depending on the participants’ gender (error bars represent 95% confidence intervals [CIs]).

**Figure 2:**
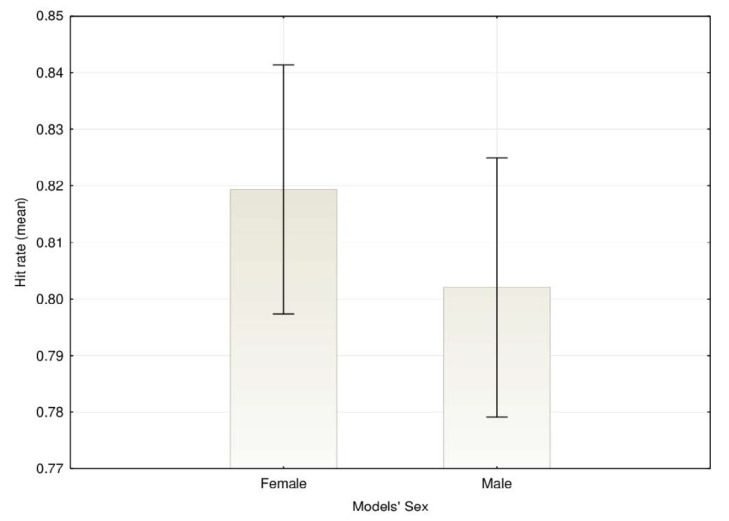
Mean agreement rates for the emotion recognition depending on the models’ sex (error bars represent 95% CI).

A main effect of participants’ gender on emotion recognition was found, *F*(1, 1244) = 21.89, *MSE* = 0.049, *p* < 0.001, η^2^_p_ = 0.017, with women presenting higher agreement rates (*M* = 82.8%, *SD* = 10.0), than men (*M* = 79.3%, *SD* = 12.2) (Figure 1). A main effect for the models’ sex was also observed, *F*(1,1244) = 27.67, *MSE* = 0.009, *p* < 0.001, η^2^_p_ = 0.022, with emotions being generally better recognized when models were women (*M* = 81.9, *SD* = 11.1) than when models were men (*M* = 80.2, *SD* = 12.0) (Figure 2). 

### 3.2. Interaction Effects on Emotion Recognition

**Figure 3:**
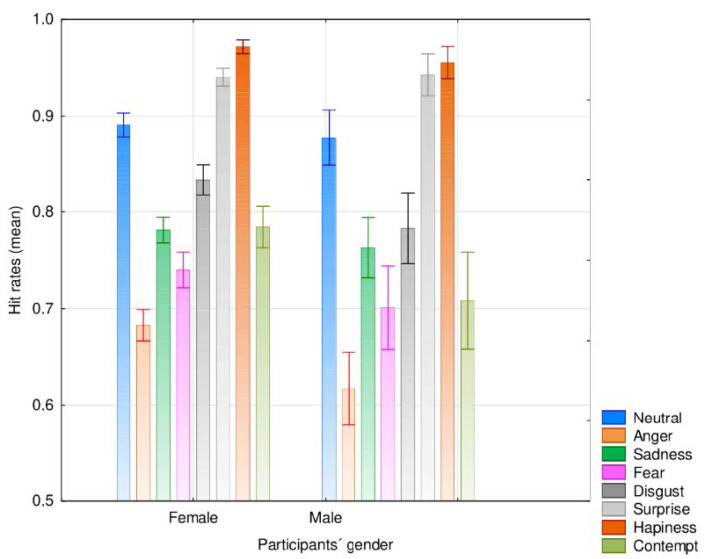
Mean agreement rates for the different emotional categories depending on the participants’ gender (error bars represent 95% CI).

The results showed an interaction effect between participants’ gender and emotional category, *F*(5.46, 6792.8) = 5.13, *MSE* = 0.030, *p* < 0.001, η^2^_p_ = 0.004. The post-hoc Unequal N HSD test showed that women presented significantly higher recognition rates than men for anger (mean agreement rate for women = 68.3%, versus men = 61.7%, *p* = 0.018) and for contempt (mean agreement rate for women = 78.4%, versus men = 70.8%, *p =* 0.001) (Figure 3). 

**Figure 4:**
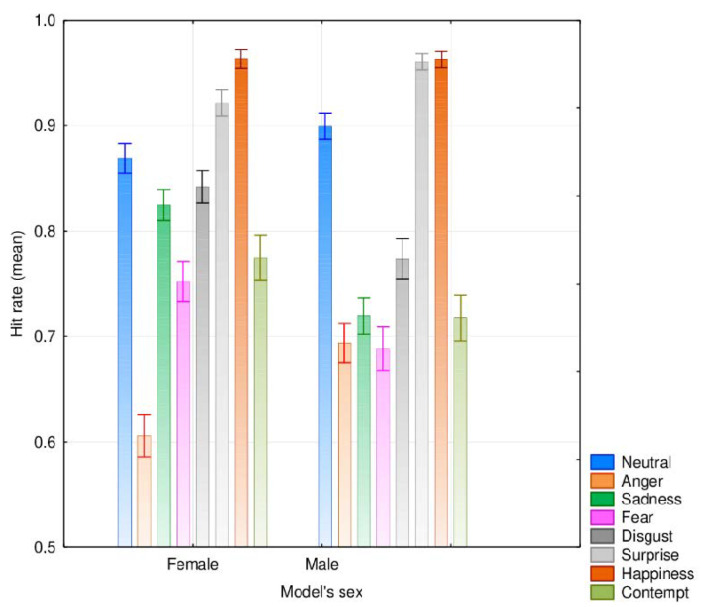
Mean agreement rates for the different emotional categories depending on the models’ sex (error bars represent 95% CI).

The analysis also showed an interaction effect between the models’ sex and emotional category, *F*(5.44, 8708) = 47.13, *MSE* = 0.030, *p* < 0.001; η^2^_p_ = 0.037. The post-hoc Tukey test showed that emotion recognition differed significantly depending on the model’s sex for all emotional categories except happiness (*p* = 1), which yielded similar and very high agreement rates in both female and male models (96.4% and 96.3%). Participants showed superior recognition of emotions in female models compared to male models for sadness (mean agreement rates of 82.5% and 72.0%, respectively, *p* < 0.001), fear (mean agreement rates of 75.2% and 68.8%, respectively, *p* < 0.001), disgust (mean agreement rates of 84.2% and 77.4%, respectively, *p* < 0.001) and contempt (mean agreement rates of 77.5% and 71.8%, respectively, *p* < 0.001). However, the agreement rates were higher in male than in female models for anger (69.4% and 60.6%, respectively, *p* < 0.001), surprise (96.1% and 92.2%, respectively, *p* < 0.001), and the neutral face (89.9% and 86.9%, respectively, *p* < 0.001; see Figure 4). The dataset is available as supplementary material (Table S1), allowing others to compute data depending on the participants’ gender and the models’ sex [66].

## 4. Discussion

In the present study, the adequacy of the RaFD as a database to be employed in emotion recognition tasks among Portuguese samples was tested. One of the goals was to present normative data regarding the recognition of seven facial expressions with emotional content, plus a neutral facial expression, utilizing a forced-choice task. The other goal was to identify differences in the recognition of facial emotional expressions according to participants’ gender and according to the sex of the model expressing the emotion.

The overall level of emotion recognition in this study was high and equivalent to that reported in the original study (82%), even if significant differences were found in the recognition of all emotions between the present study and the study by Langner et al. [32]. This shows that the RaFD is an appropriate resource for studying emotion recognition among Portuguese samples, or at least Portuguese college students. The results also show that some facial expressions were more easily recognized than others. As in the original study, positive emotions were more easily recognized than negative ones [32]. More specifically, happiness was the most easily recognized emotion, with a high percentage of correct responses (97%), as found in previous literature [67,68,69]. The least recognized facial expression was anger (69%), while contempt showed the lowest recognition rates (53%) in the original study [31]. 

Although some studies point to the interaction between cognition and emotion as a possible explanation for the differences found between emotions of positive and negative valence [13,39,40,41,43,44,45], a simpler explanation is that there are no alternative responses of positive valence competing with happiness. However, there are several ones competing with each of the negative emotions. Indeed, previous findings e.g., [70] show that confusion between emotions of negative valence (e.g., anger and disgust, or fear and sadness) is much higher than between happiness and any negative emotion. Otherwise, the lower ability to recognize emotions of negative valence, such as angry facial expressions, would not make sense from a functional-evolutionary point of view. Disgust was the most recognized of the negative facial expressions of emotion, which is consistent with previous literature [32]. 

Considering the participants’ gender, differences between sexes were only significant for anger and contempt (two of the three least recognized emotions in the present sample). The (small) advantage in favor of women on emotion recognition, moderated by factors such as the specific emotions, emotion type, sex of the actor, among others, has been reported in the literature (see [56] for a review). Moreover, other variables could operate underneath. For example, a recent study has shown an interaction between gender and hometown [71].

Regarding the model’s sex, statistically significant differences in recognition rates emerged for most expressions. Only happiness yielded similar results in male and female models. Sadness, fear, disgust, and contempt were more recognizable in female compared to male models, whereas anger, surprise and the neutral face were more recognizable in male models. Langner et al. (2010) found that unbiased “hit-rates” were higher for female than male models, and post hoc tests showed significantly higher agreement rates for happiness and lower hit rates for contempt [32]. Additionally, Calvo and Lundqvist (2008) found a tendency for angry faces to be better recognized in male than in female models [72].

The present study has some potential limitations. For instance, it did not measure the arousal of emotional faces, and there are arousal effects that have been found in the perception of emotional information in different cultures [73]. This should be considered in future studies. As in the study by Langner et al. (2010), the sample of the present study comprised a higher number of female participants, corresponding to the typical gender distribution in health sciences courses in Portuguese universities [32]. The lack of balance regarding participants’ gender is not optimal when examining sex differences in the emotion recognition. However, a sizable number of men still participated in this study. In addition, unlike previous research e.g., [55], the present study examined all emotions available in the RaFD database. Happiness was the only positive emotion present in it (though surprise can have both a positive and negative valence), as in most stimuli sets of facial expressions of emotion.

Furthermore, besides communicating emotions, some authors have argued that our facial expressions are used to influence others [74,75,76]. Taking into account this perspective, naturalistic studies are emerging as an alternative approach to the study of emotions and its relevance is increasing e.g., [77].

Despite the aforementioned limitations, further studies would be useful to investigate other parameters available in the RaFD that were not considered here, such as child models, gaze directions (other than direct gaze), and other camera angles. Similarly, more research is needed to explore whether subclinical and clinical samples demonstrate particular difficulties recognizing facial expressions of emotion in comparison to the sample of this study. These aspects are important to ascertain the utility of this material and these data, including for research and clinical application. 

## 5. Conclusions

Most studies on the recognition of emotional expressions resort to the presentation of static pictures or slides of faces. The RaFD provides adequate stimuli for studies involving the recognition of emotional facial expressions. It can now be applied (in Portugal) for research in this field, namely in areas such as neurobiological research, clinical practice, education, and justice, at least among university students. Such applications require the development of sets of standardized stimuli and their validation, including normative data for different countries and cultures. This study (and the information provided in the supplementary material) contributes to this goal and thus to the possibility of further research toward progress regarding our understanding of this topic. 

The results show high levels of recognition of the facial expressions and are similar to those obtained in other studies using the RaFD, including the original study. Given that the validation data are available online, researchers can select the most appropriate stimuli for their research.

The influence of participants’ gender on recognition levels was limited to just two emotions (anger and contempt). However, the fact that the sex of the models in the pictures affected emotion recognition suggests that the selection of men or women for the display of specific emotions requires further research on this topic.

## Figures and Tables

**Table 1:** Mean and standard deviation (*SD*) of agreement and mean unbiased “hit-rates” per expression (%).

Agreement	Perceived Emotion							
	Neutral *M* *(SD)*	Anger *M* *(SD)*	Sadness *M* *(SD)*	Fear *M* *(SD)*	Disgust *M* *(SD)*	Surprise *M* *(SD)*	Happiness *M* *(SD)*	Contempt *M* *(SD)*
Depictedemotion								
**Anger**	4.9 (7.8)	67.7 (25.3)	7.9 (12.8)	2.4 (2.8)	3.3 (4.4)	3.9 (5.0)	0.2 (0.37)	8.4 (7.4)
**Sadness**	6.6 (15.0)	2.4 (4.7)	76.2 (24.2)	2.7 (3.1)	2.1 (4.0)	1.1 (1.7)	0.2 (0.3)	8.1 (11.2)
**Fear**	0.6 (1.5)	1.2 (1.2)	1.0 (1.5)	73.8 (12.6)	6.5 (8.6)	15.1 (11.8)	0.3 (0.5)	0.9 (1.3)
**Disgust**	0.4 (0.4)	9.4 (11.8)	0.4 (0.5)	0.8 (0.6)	80.9 (12.5)	2.2 (3.2)	0.2 (0.3)	5.3 (2.9)
**Surprise**	0.4 (0.4)	0.2 (0.3)	0.2 (0.3)	2.5 (2.8)	0.8 (1.3)	94.2 (3.9)	0.4 (0.5)	1.0 (1.8)
**Happiness**	0.6 (0.7)	0.2 (0.3)	0.3 (0.4)	0.2 (0.3)	0.3 (0.4)	0.8 (0.7)	96.8 (1.6)	0.5 (0.6)
**Contempt**	12.6 (10.6)	0.8 (1.3)	3.7 (2.4)	0.4 (0.5)	1.2 (0.7)	0.7 (0.7)	1.9 (3.9)	78.1 (11.8)

Note: This table can also be interpreted in terms of a ‘confusion matrix’ (darker grey indicates either higher ‘hit rates’ or higher confusion between the displayed and perceived emotion).

**Table 2:** Comparison between emotion recognition (mean agreement rates) in this study (*N* = 1249) and in the Langner et al.´s (2010) study (*N* = 276).

	Dores et al., 2020 [66]		Langner et al., 2010 [32]			
	*M*	*SD*	*M*	*SD*	*t*	*df*
**Neutral**	88.3	9.7	83.0	13.0	7.65 ***	345
**Anger**	67.7	25.0	81.0	19.0	8.32 ***	509
**Sadness**	76.2	24.0	85.9	16.0	6.41 ***	584
**Fear**	73.4	13.0	88.0	7.0	18.04 ***	757
**Disgust**	80.9	12.0	79.0	10.0	2.45 *	467
**Surprise**	94.2	4.0	90.0	9.0	11.99 ***	299
**Happiness**	96.8	2.0	98.0	3.0	8.15 ***	330
**Contempt**	78.1	12.0	48.0	12.0	37.71 ***	1523

Note: * *p* < 0.05, *** *p* < 0.001, *t* = *t*-test

## Supplementary Materials

The following are available online at https://www.mdpi.com/1660-4601/17/20/7420/s1, Table S1: Portuguese normative data of the Radboud Faces Database.
